# Mechanisms of wheat (*Triticum aestivum*) grain storage proteins in response to nitrogen application and its impacts on processing quality

**DOI:** 10.1038/s41598-018-30451-4

**Published:** 2018-08-09

**Authors:** Ting Zheng, Peng-Fei Qi, Yong-Li Cao, Ya-Nan Han, Hong-Liang Ma, Zhen-Ru Guo, Yan Wang, Yuan-Yuan Qiao, Shi-Yu Hua, Hai-Yue Yu, Jiang-Ping Wang, Jing Zhu, Cai-Yi Zhou, Ya-Zhou Zhang, Qing Chen, Li Kong, Ji-Rui Wang, Qian-Tao Jiang, Ze-Hong Yan, Xiu-Jin Lan, Gao-Qiong Fan, Yu-Ming Wei, You-Liang Zheng

**Affiliations:** 1 Triticeae Research Institute, Sichuan Agricultural University, Chengdu, Sichuan 611130 China; 2 Agronomy College, Sichuan Agricultural University, Chengdu, Sichuan 611130 China

## Abstract

Basis for the effects of nitrogen (N) on wheat grain storage proteins (GSPs) and on the establishment of processing quality are far from clear. The response of GSPs and processing quality parameters to four N levels of four common wheat cultivars were investigated at two sites over two growing seasons. Except gluten index (GI), processing quality parameters as well as GSPs quantities were remarkably improved by increasing N level. N level explained 4.2~59.2% and 10.4~80.0% variability in GSPs fractions and processing quality parameters, respectively. The amount of N remobilized from vegetative organs except spike was significantly increased when enhancing N application. GSPs fractions and processing quality parameters except GI were only highly and positively correlated with the amount of N remobilized from stem with sheath. N reassimilation in grain was remarkably strengthened by the elevated activity and expression level of glutamine synthetase. Transcriptome analysis showed the molecular mechanism of seeds in response to N levels during 10~35 days post anthesis. Collectively, we provided comprehensive understanding of N-responding mechanisms with respect to wheat processing quality from N source to GSPs biosynthesis at the agronomic, physiological and molecular levels, and screened candidate genes for quality breeding.

## Introduction

Wheat is one of the major cereal crops in the world, and provides an important source of vegetable protein for human beings. Compared to rice and corn, wheat is the source of numerous foods depending on the unique properties of its grain storage proteins (GSPs)^1^. The quantity and composition of GSPs are associated with processing and nutritional quality of wheat, and thus affect wheat price in essential market.

GSPs in wheat, mainly referring to gliadins and glutenins, together accounting for 60~80% of the total seed proteins, are important determinants of processing quality, as they are responsible for the elasticity and extensibility of dough that determine the processing qualities of various end-products^2^. Gliadins are monomeric, and have been divided into three classes, i.e. α/β-, γ-, and ω-gliadins. Glutenins are polymeric fractions composed of high-molecular-weight (HMW-GSs) and low-molecular-weight glutenin subunits (LMW-GSs) linked with intermolecular disulfide bonds^3^.

In wheat, 60~95% of the grain nitrogen (N), which is principally used for GSPs synthesis, derives from the remobilization of N stored in vegetative organs before anthesis, particularly in leaf blades, stem and sheaths^4,5^. During senescence after anthesis, proteinaceous components of vegetative organ cells are degraded into amino acids, amides and ammonium^6^. The major part of ammonium is incorporated into amino acids for export by glutamine synthetase (GS) and glutamate synthase (GOGAT) cycle^7^. Subsequently, amino acids as the major N-transport compound are exported via the phloem into developing grains to synthesize GSPs. N remobilization is closely correlated to the synthesis of GSPs^8^, while there is little imformation on the relationship between N remobilization and GSPs synthesis in wheat. In addition, a large number of studies focus on GS enzyme in leaf due to its major importance for the re-assimilation of ammonium into exported amino acids during senescence^7,9,10^, whereas its function in developing seeds is usually ignored.

N is one of the curial macronutrients for plant growth. It is generally accepted that N application, as an important agronomic input, plays a vital role in the well balance of yield and processing quality in wheat production, by delaying vegetative senescence and increasing the amount of N reserve at anthesis^11,12^. N remobilization is greatly strengthened by the elevated GS activity under abundant N level^13,14^. Adequate N supply exerts a significant increase in protein content and dough quality^15,16^. To date, researchers have investigated the response of protein accumulation during grain development of wheat to varied N by transcriptome, proteomics and metabolite profiling methods^15,17,18^. However, very little information covers the entire grain filling stage and exclusively illustrates physiological, biochemical and molecular changes in GSPs biosynthesis under sufficient N supply. Our knowledge of the underlying basis for the establishment of processing quality after N application remains fragmentary.

Here, we performed field trials with four common wheat cultivars under four N treatments at two sites over two wheat growing seasons. Transcriptome profiling in developing grain was combined with the phenotypic and physiological characters, such as the accumulation of GSPs fractions, processing quality parameters, the status of N remobilization in wheat plants, and dynamic enzyme activities related to N reassimilation. This research would deepen our understanding on the mechanisms of the establishment of wheat processing quality in response to N application. In addition, the identified genes would be important candidates for wheat quality breeding in the future.

## Results

### Processing quality parameters

Processing quality parameters of the four cultivars shown a similar trend after increasing N application at two sites in two consecutive growing seasons (Table 1). GPC, SV, GC, GI, DT and ST were significantly influenced by N level and genotype at both sites in two growing seasons. The percentages of variability of GPC, SV, GC, DT and ST explained by N level accounted for 57.8~80.0%, 23.3~72.6%, 37.0~63.7%, 16.2~41.9% and 10.4~31.6%, respectively, while that of GI was only 3.8~16.0%, indicating that GI was mainly controlled by genotype. Only DT and ST showed significant N level × genotype interaction at both sites in two growing seasons, and the percentages of variability of DT and ST explained by N level × genotype interaction were much lower than that explained by N level or genotype. To summarize, GPC, SV, GC, DT and ST of four cultivars were statistically improved when increasing N level, whereas GI was decreased by 5.3~14.0%, especially in 2015–2016 growing season.

**Table 1:** Processing quality parameters of four cultivars grown at Chongzhou and Renshou under different N Levels in 2014–2015 and 2015–2016 growing seasons.

N level	Chongzhou						Renshou					
	GPC	SV	GC	GI	DT	ST	GPC	SV	GC	GI	DT	ST
**2014–2015 growing season**												
Mean												
N_0_	10.20d	11.6d	18.7d	69.8a	1.18d	1.55c	11.39d	20.8d	21.6d	84.2a	1.34c	2.47c
N_75_	11.67c	19.1c	24.1c	70.6a	1.55c	2.44b	11.95c	24.2c	23.3c	82.3a	1.72c	3.61b
N_150_	13.32b	24.7b	28.9b	64.5b	2.15b	2.61ab	12.74b	26.6b	25.7b	81.3ab	2.37b	4.04ab
N_225_	13.97a	26.8a	29.9a	67.5ab	2.39a	2.76a	13.76a	29.8a	28.6a	78.0b	3.26a	4.61a
Percentage of variance explained												
Genotype	13.2	26.2	30.6	56.9	34.0	61.0	26.9	62.9	42.3	77.2	24.6	52.7
N	80.0	64.8	63.7	7.6	41.9	17.7	53.8	23.3	37.0	3.8	33.3	10.4
G × N	2.8	5.2	3.1	8.7	19.4	13.1	4.2	6.0	2.4	2.9	26.1	15.0
F-value												
Genotype	52.2**	48.6**	221.5**	30.6**	92.2**	104.3**	23.8**	97.6**	23.9**	18.4**	20.1**	13.1**
N	190.0**	235.8**	377.9**	4.4*	91.9**	40.3**	72.0**	51.4**	34.3**	4.3*	22.2**	8.3**
G × N	2.3	6.3**	6.2**	1.7	14.2**	10.0**	1.9	4.4**	0.8	1.1	5.8**	4.0**
**2015–2016 growing season**												
Mean												
N_0_	9.50d	9.5d	15.8d	82.4a	0.99c	1.38b	9.90d	14.4d	19.1d	86.6a	1.02b	1.81b
N_75_	10.17c	12.0c	18.2c	75.5b	0.98c	1.47b	10.50c	16.6c	21.1c	83.7ab	1.15b	1.96b
N_150_	11.06b	15.7b	20.6b	73.6b	1.14b	1.96a	11.35b	19.2b	23.2b	78.6b	1.34a	2.12b
N_225_	12.22a	18.9a	24.1a	70.6b	1.40a	2.18a	12.66a	21.7a	26.4a	72.7c	1.41a	3.06a
Percentage of variance explained												
Genotype	26.7	12.5	41.6	78.8	18.5	35.6	25.1	35.3	43.3	52.8	36.1	19.4
N	57.8	72.6	46.6	6.3	28.0	28.2	56.8	45.6	37.3	16.0	16.2	31.6
G × N	2.8	5.5	2.2	4.3	32.7	17.1	3.6	2.5	1.8	3.2	22.9	23.9
F-value												
Genotype	13.4**	8.5*	27.4**	107.4**	4.7	36.4**	6.6*	8.9*	8.9*	11.0**	10.6**	7.7*
N	88.7**	119.3**	72.9**	6.0**	21.5**	13.3**	133.4**	84.9**	56.1**	10.1**	8.0**	12.7**
G × N	1.4	3.0*	1.2	1.4	8.4**	2.7*	2.9*	1.5	0.9	0.7	3.7**	3.2*

GPC, grain protein content; SV, zeleny sedimentation value; GC, wet gluten content; GI, gluten index; DT, development time; ST, stable time; G × N, interaction between N level and genotype. Different letters after numbers indicate significance at *P* < 0.05. “*” and “**” after F-values represent significant difference at *P* < 0.05 and *P* < 0.01, respectively.

### Content and composition of glutenin and gliadin fractions in flour

The content and composition of glutenin and gliadin fractions were significantly affected by N level and genotype but not the N level × genotype interaction (Table 2). Genotype contributed more variance to the content and composition of glutenin and gliadin fractions than N level, indicating the diversity of cultivars used in this experiment. The application of N significantly promoted the accumulation of glutenin and gliadin fractions, and the percentage was highest for HMW-GSs from N_0_ to N_225_ treatment (86.6% at Chongzhou and 50.7% at Renshou), followed by LMW-GSs, α/β-gliadin, γ-gliadin and ω-gliadin. Notably, Glu/Gli was decreased when increasing N level.

**Table 2:** Content and composition of glutenin and gliadin fractions for four cultivars grown at Chongzhou and Renshou under four N levels in 2014–2015 growing season.

	HMW	LMW	H/L	Glutenins	ω-gliadin	α/β-gliadin	γ-gliadin	Gliadins	ω-gliadin %	α/β-gliadin %	γ-gliadin%	Glu/Gli
**Chongzhou**												
Mean												
N_0_	3.82c	8.34c	0.52b	12.16c	6.77d	20.37b	10.00c	37.15c	17.87a	55.31	26.82b	1.10a
N_75_	5.13b	10.67b	0.52ab	15.79b	8.14c	24.53b	12.90b	45.56b	17.40ab	54.37	28.23a	1.06a
N_150_	6.92a	13.66a	0.53ab	20.58a	9.71b	30.38a	15.39ab	55.48a	17.11ab	55.19	27.70ab	0.97b
N_225_	7.13a	13.59a	0.55a	20.72a	10.01a	34.09a	17.98a	62.09a	16.59b	55.11	28.30a	0.96b
Percentage of variance explained												
Genotype	48.1	25.4	83.1	22.5	84.8	19.0	31.9	29.2	95.2	90.5	62.8	15.4
N	37.7	43.2	0.3	59.2	12.6	43.6	31.2	40.9	1.0	0.8	5.5	38.7
G × N	5.4	3.3	1.5	5.1	1.9	10.6	12.6	9.1	0.9	2.8	11.6	10.8
F-value												
Genotype	33.9**	44.0**	60.4**	11.3**	1008.4**	8.5*	22.9**	19.6**	359.9**	273.7**	14.6**	13.7**
N	54.2**	49.1**	2.0	53.4**	273.1**	18.2**	12.5**	21.2*	3.3*	1.3	2.3	17.0**
G × N	2.6*	1.2	3.2*	1.5	13.5**	1.5	1.7	1.6	1.1	1.5	1.7	1.6
**Renshou**												
Mean												
N_0_	5.42d	11.73c	0.51c	17.15d	8.08c	23.27c	12.35c	43.70c	17.89a	53.88	28.23	1.12a
N_75_	6.06c	12.74bc	0.54b	18.80c	8.28c	24.35c	12.61c	45.24c	17.92a	54.46	27.62	1.10a
N_150_	6.91b	13.60b	0.56ab	20.51b	9.03b	27.04b	14.04b	50.12b	17.73ab	54.45	27.83	1.11a
N_225_	8.17a	15.47a	0.58a	23.64a	9.95a	30.08a	15.88a	55.91a	17.45b	54.29	28.26	1.05b
Percentage of variance explained by												
Genotype	46.6	62.8	78.4	50.2	92.6	39.0	67.4	61.2	98.6	92.6	81.5	30.1
N	29.9	11.1	1.1	20.6	4.2	42.6	17.2	25.2	0.2	0.3	0.7	16.5
G × N	2.4	0.7	0.9	0.6	0.6	3.5	1.6	1.8	0.2	0.7	2.4	12.0
F-value												
Genotype	10.0**	34.6**	17.0**	16.2**	205.3**	21.5**	52.9**	44.6**	892.0**	149.1*	61.5**	4.8*
N	38.7**	18.4**	8.5**	27.2**	30.8**	56.0**	21.7**	42.3**	2.2	0.6	0.6	5.4**
G × N	1.0	0.4	2.5*	0.3	1.4	1.5	0.7	1.0	1.0	0.4	0.6	1.3

Values of the amount of glutenin and gliadin fractions are shown as 10^3^ absorbance units (abbreviated as mAU) of RP-HPLC corresponding to 1 mg of flour. LMW, low-molecular-weight glutenin subunits; HMW, high-molecular-weight glutenin subunits; H/L, ratio of HMW to LMW; %, % total gliadins; Glu/Gli, ratio of glutenins to gliadins. Different letters after numbers indicate significance at *P* < 0.05. “*” and “**” after F-values represent significant difference at *P* < 0.05 and *P* < 0.01, respectively.

**Figure 1:**
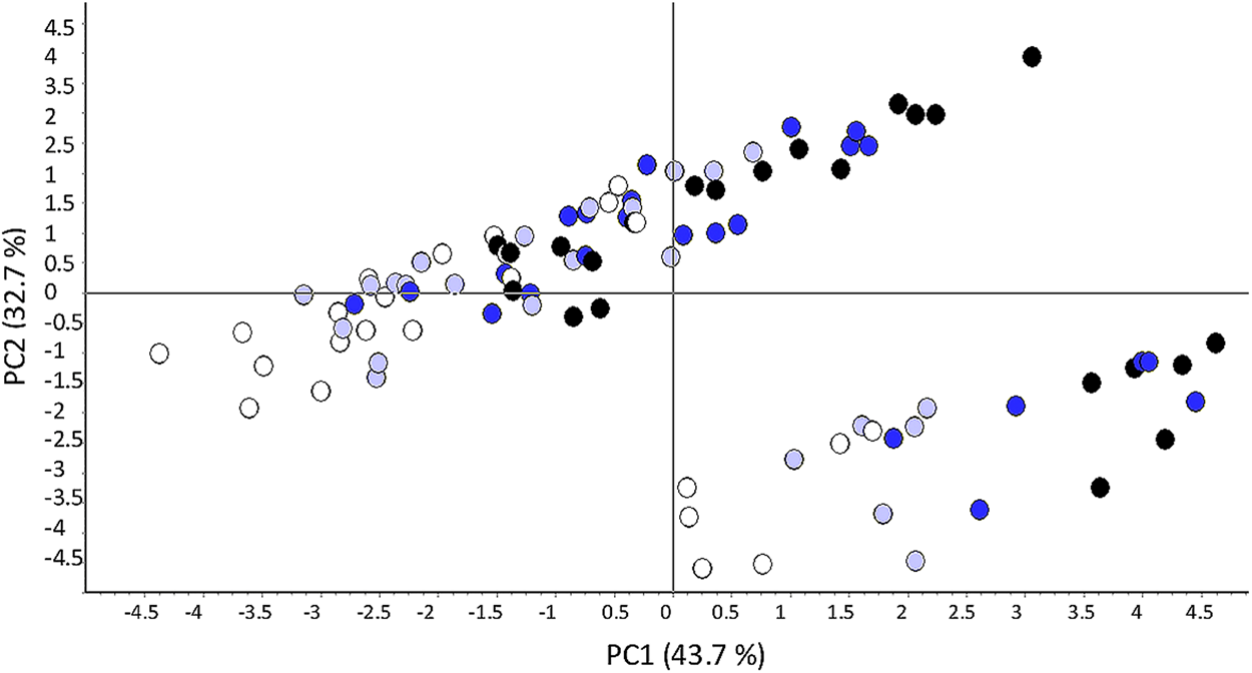
Coordinates for samples determined by RP-HPLC for the first two PCs showing the separation of samples according to N levels. White, light blue, dark blue and black cakes indicate the N_0_, N_75_, N_150_ and N_225_ treatments, respectively.

RP-HPLC data were analyzed by principal component analysis. The first four principal components (PCs) accounted for 94.1% of the variation in the distance matrix formed from the similarities between samples (Supplementary Table S1). PC1 and PC2 covered 43.7% and 32.7% of the total variation, respectively, indicating that the first two PCs could explain the variation of protein fractions under differed N levels. PC1 axis mainly depended on total gliadins, HMW-GSs, γ-gliadin, ω-gliadin and α/β-gliadin. LMW-GSs, ω-gliadin%, and total glutenins were important for PC2 axis. The four cultivars were gathered in two groups. Meanwhile, the four N levels were clearly separated from each other. The higher N levels had a trend of migration from negative axis to positive axis, and the migrated distance was positively associated with N level (Fig. 1).

### N remobilization from vegetative organs

**Figure 2:**
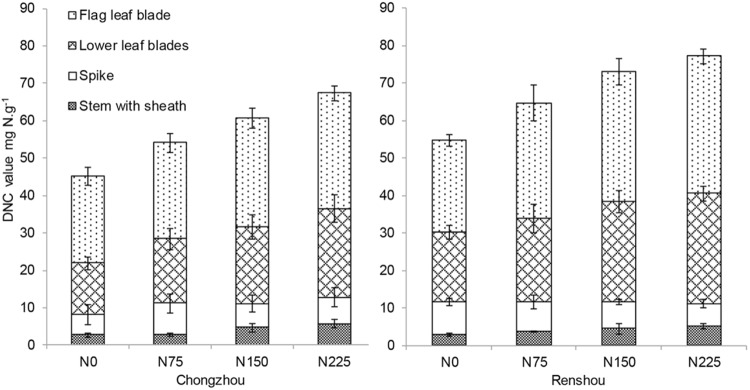
DNC (difference of N concentration) in vegetative organs between anthesis and maturity, under four N treatments during 2014–2015 growing season.

The difference of N concentration (DNC) between maturity and anthesis was highest in flag leaf blade, and then in the lower leaf blades, followed in spike and stem with sheath (Fig. 2). Significant differences were identified in the amount of N remobilized from vegetative organs, among four N levels for four cultivars. The amount of remobilized N at Renshou was higher than that at Chongzhou. DNC in flag leaf blade, the lower leaf blades and stem with sheath were significantly increased with the elevated N application at both sites, while DNC in spike differed between both sites, and even decreased at Renshou.

### Correlations among processing quality parameters, content and composition of GSPs, and N remobilization

Significant and varied correlations were broadly observed between content and composition of GSPs and processing quality parameters (Table 3). For processing quality parameters, GI and GC had a stronger correlation with the composition than the content of GSPs. For the composition of glutenin and gliadin fractions, γ-gliadin% and Glu/Gli were significantly associated with all processing quality parameters.

**Table 3:** Correlation coefficients between content and composition of glutenin and gliadin fractions and wheat processing quality parameters.

Traits	GPC	SV	GC	GI	DT	ST
HMW	0.738**	0.665**	0.745**	−0.182	0.660**	0.499**
LMW	0.398**	0.649**	0.357**	0.420**	0.377**	0.527**
H/L	0.312**	0.048	0.344**	−0.526**	0.245*	−0.012
Glutenins	0.600**	0.758**	0.571**	0.241*	0.552**	0.599**
ω-gliadin	0.480**	0.138	0.482**	−0.606**	0.338**	−0.016
α/β-gliadin	0.802**	0.692**	0.803**	−0.253*	0.661**	0.462**
γ-gliadin	0.713**	0.713**	0.730**	−0.163	0.661**	0.546**
Gliadins	0.802**	0.646**	0.809**	−0.363**	0.670**	0.425**
ω-gliadin %	0.033	−0.297**	0.012	−0.539**	−0.030	−0.319**
α/β-gliadin %	−0.197	−0.062	−0.205*	0.372**	−0.196	−0.042
γ-gliadin %	0.235*	0.567**	0.279**	0.323**	0.334**	0.573**
Glu/Gli	−0.544**	−0.243*	−0.570**	0.519**	−0.344**	−0.013

Abbreviations are used as in Tables 1 and 2. “*” and “**” represent significance at *P* < 0.05 and *P* < 0.01, respectively.

DNC in stem with sheath displayed the highest correlation with processing quality parameters and with the content and composition of GSPs (Table 4). DNC in stem with sheath was significantly and positively correlated with GPC, LMW-GSs, ω-gliadin and GC, while significantly and negatively correlated with GI (Table 4). It suggested that N remobilized from stem with sheath was critical for the accumulation of LMW-GSs and ω-gliadin, resulting in increased GPC in grain and altered processing quality. In addition, GI was the only one which had high correlation with DNC in vegetative organ in all above processing quality parameters. Extremely significant and positive correlation was found between GI and DNC in spike, whereas negative correlation was detected between GI and DNC in leaf, stem and sheath.

**Table 4:** Correlation coefficients for relation between DNC (difference of N concentration between maturity and anthesis) in vegetative tissues and RP-HPLC data & processing quality parameters.

Traits	Stem with sheath	Spike	Lower leaf blades	Flag leaf blade
GPC	0.501*	0.089	0.273	0.079
HMW	0.233	−0.151	0.009	0.048
LMW	0.430*	−0.028	0.104	0.125
H/L	−0.206	−0.111	−0.042	−0.128
Glutenins	0.368	−0.074	0.072	0.099
ω-gliadin	0.456*	0.216	0.299	0.046
α/β-gliadin	0.122	−0.077	−0.033	−0.094
γ-gliadin	0.077	−0.034	−0.015	−0.11
Gliadins	0.128	−0.046	−0.008	−0.095
ω-gliadin%	−0.017	0.143	0.043	0.123
α/β-gliadin%	0.047	−0.442*	−0.177	0.093
γ-gliadin%	−0.031	0.3	0.129	−0.16
Glu/Gli	−0.140	0.022	0.152	0.218
SV	0.306	0.004	0.04	0.016
GC	0.453*	0.185	0.159	−0.006
GI	−0.432*	0.520**	−0.304	−0.448*
DT	0.193	−0.348	0.141	0.291
ST	0.106	−0.26	−0.101	0.158

Abbreviations were used as Tables 1 and 2. “*” represents significance at *P* < 0.05.

### Enzyme activity of GS

**Figure 3:**
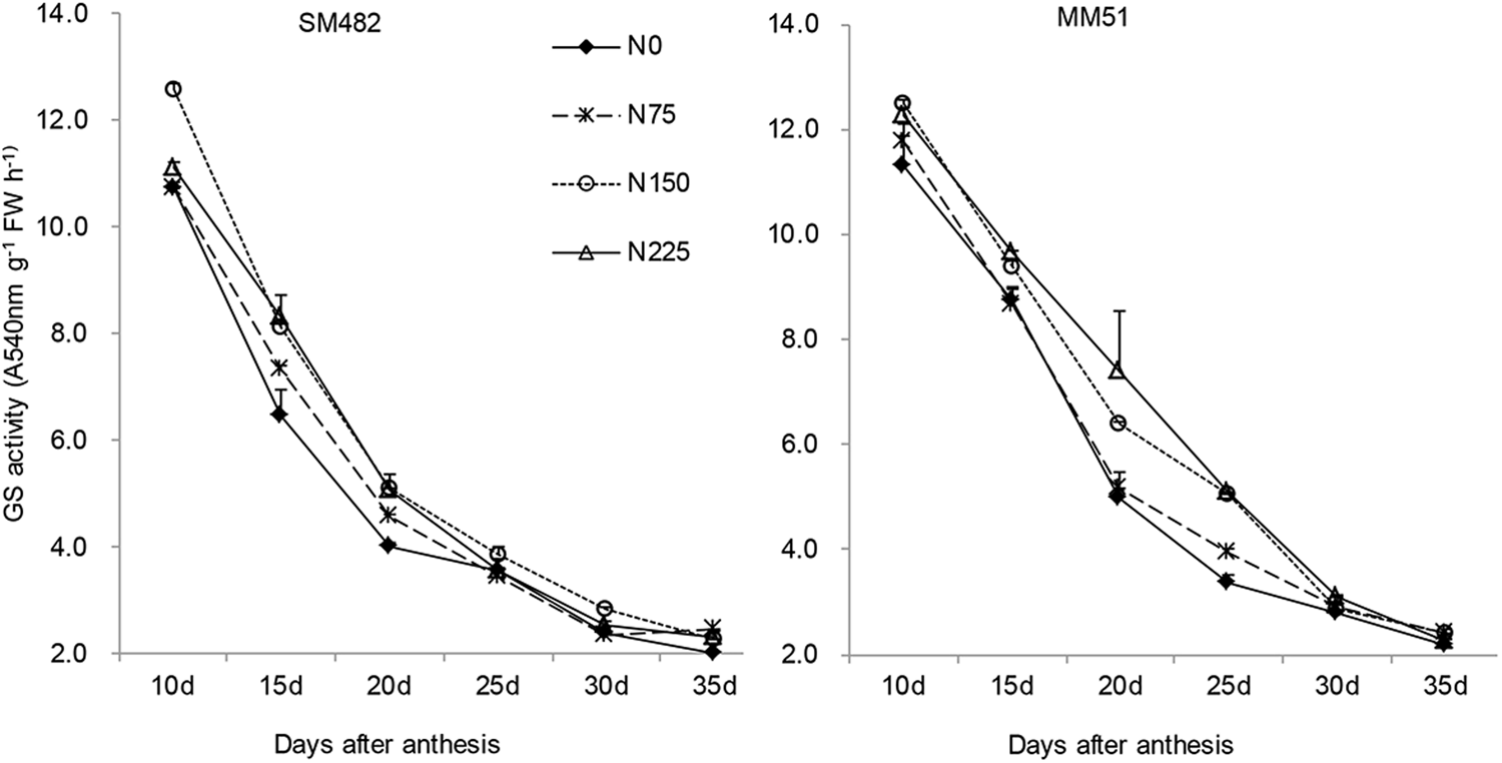
GS activities in grains of SM482 and MM51 at Chongzhou during 2014–2015 growing season.

**Figure 4:**
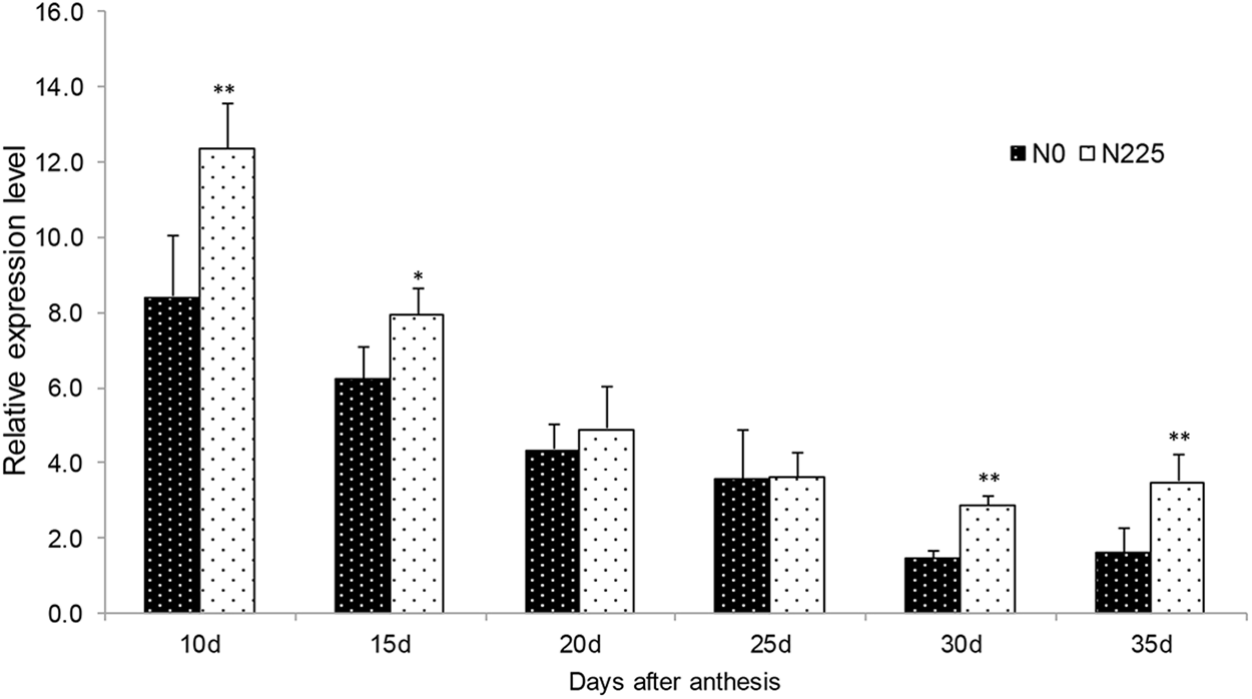
Expression of *GSr2* in developing grains of SM482 at Chongzhou in 2014–2015 growing season. “*” and “**” indicate significant difference between N_0_ and N_225_ at 5% and 1% levels, respectively.

GS activities in grains exhibited rapidly decreasing trends during 10~35 DAA, especially during 10~20 DAA (Fig. 3). GS activities of SM482 and MM51 under N_150_ and N_225_ treatments were significantly higher than those under N_0_ and N_75_ treatments at most time points. It suggested that increased N application was conducive to improve GS activities in grains, which was supported by RNA-seq and qRT-PCR (Fig. 4). Transcriptome analysis (N_0_ VS N_225_) revealed that three *GS* transcripts, one encoding *GSr1* and two encoding *GSr2* isoforms, were always up-regulated (Log2 > 0.7) with high expression level during grain filling stage.

### KEGG and GO enrichment analysis

There were 2293 DEGs (N_0_ VS N_225_) screened during 10~35 DAA (Supplementary Table S2), with 158 DEGs being detected at more than one time point (Supplementary Table S2B). To validate the expression profiles obtained by RNA-seq, qRT-PCR was performed to measure the expression of 10 DEGs (with high fold changes, with high expression level (FPKM > 100), and in starch or protein biosynthesis) during 10~35 DAA. As a result, a high correlation (R^2^ = 0.818) was identified between qRT-PCR and RNA-seq data, confirming the validity of RNA-seq data (Supplementary Fig. S1).

DEGs were enriched in 78 KEGG pathways (Supplementary Table S3A), and 60 pathways were found at more than one time point. Seven pathways, including photosynthesis, galactose metabolism, starch and sucrose metabolism, protein processing in endoplasmic reticulum, amino sugar and nucleotide sugar metabolism, phenylpropanoid biosynthesis and ribosome, showed enrichment during the whole filling stage. Importantly, protein processing in endoplasmic reticulum, which was highly correlated with GSPs synthesis, displayed significant enrichment during 15~25 DAA (*P* < 0.05). Eight, fifteen, thirteen and seventeen pathways were found enrichment at 5, 4, 3 and 2 time points, respectively. Nineteen pathways in Supplementary Table S3B might directly related to GSPs biosynthesis, due to the presence of key words, such as protein, amino acid, RNA, DNA, ribosome, nitrogen, phagosome and N-glycan.

**Figure 5:**
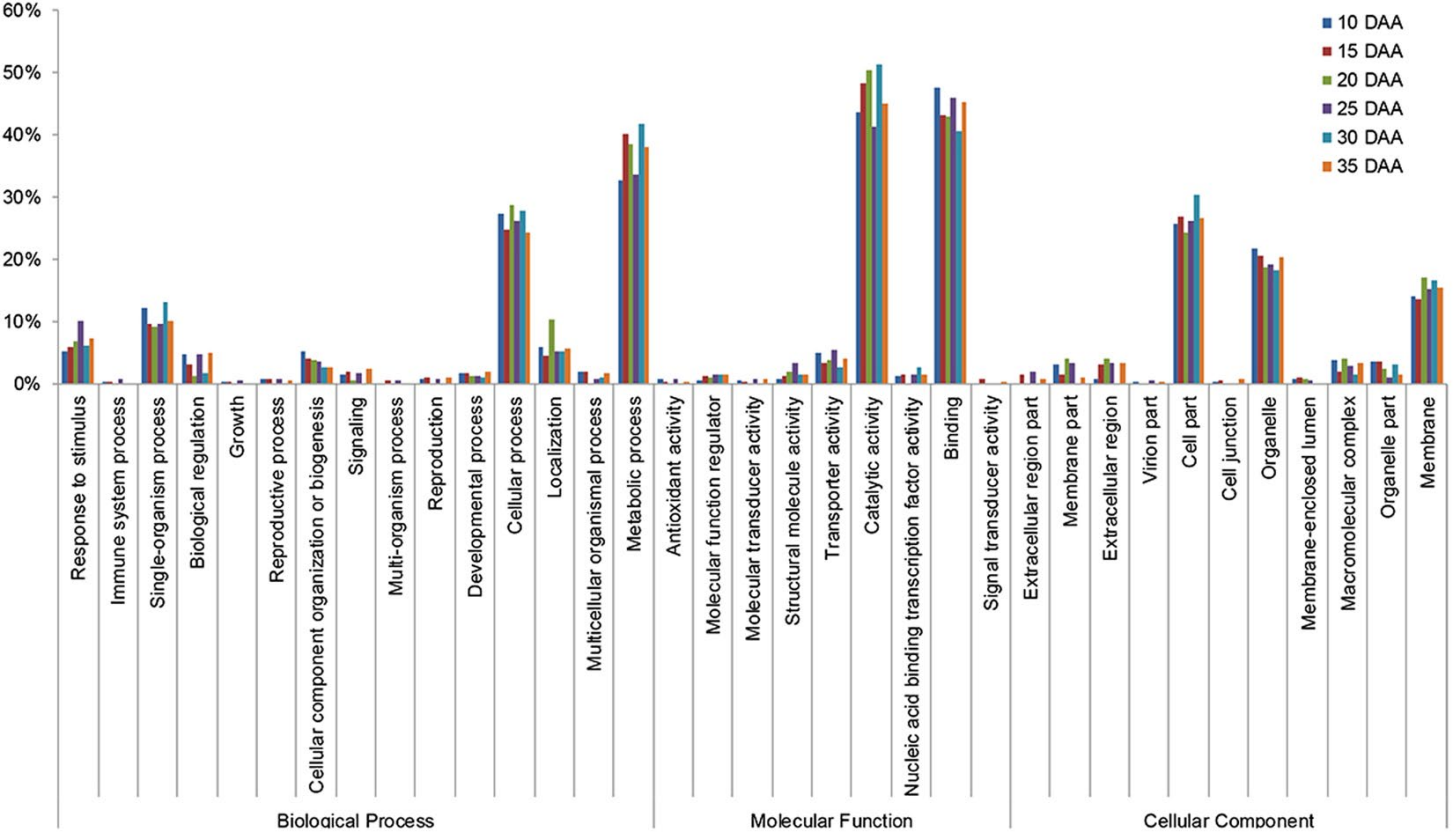
GO enrichment analysis.

GO functional enrichment analysis was performed as well (Fig. 5). According to molecular function, DEGs were mainly mapped to catalytic activity, binding and transporter activity, accounting for 43.7~51.4%, 40.5~47.5%, and 2.7~5.3% of the total DEGs detected during 10~35 DAA, respectively. According to biological process, DEGs were mainly mapped to metabolic process, cellular process, single-organism process, response to stimulus, accounting for 32.8~41.7%, 24.4~28.7%, 9.2~13.0%, and 5.2~10.0%, respectively. According to cellular component, DEGs were mainly mapped to cell part, organelle, and membrane, accounting for 24.4~30.3%, 18.2~21.8%, and 13.5~17.1%, respectively. Specifically, DEGs were enriched in 589, 78, and 169 GO terms in the biological processes, cellular components, and molecular function categories, respectively, during 10~35 DAA (Supplementary Table S4A,B and C). Ninety-four GO terms, including key words, such as nitrogen compound, transcription, amino acid, protein, ribosome, translational, gene expression, plastid, preribosome, ribosome, peptide, endoplasmic reticulum, might be involved in GSPs biosynthesis (Supplementary Table S4D).

### DEGs involved in amino acid metabolism

DEGs related to grain amino acid metabolism were mainly observed during 25~35 DAA. Genes associated with alanine, aspartate and glutamate metabolism exhibited the most dominant changes under N_225_ treatment (Supplementary Table S5). Alanine and glutamate metabolism were strengthened by the up-regulation of alanine aminotransferase 2 and *GSr1*, *GSr2* and *GSr3*, respectively, under N_225_ treatment. However, aspartate metabolism was diminished by the three down-regulated DEGs encoding asparaginase. Five DEGs involved in phenylalanine, tyrosine and tryptophan biosynthesis were upregulated as well. No DEG involved in the metabolism of other amino acids was identified.

### DEGs encoding GSPs

The expression of GSPs genes exhibited an increasing trend from 10 DAA, and reached the maximum during 30~35 DAA (Table 5). Ten DEGs encoding globulins were detected, six of which were down regulated (FDR < 0.05). Twelve DEGs encoding glutenins and gliadins were detected, and most of them were up-regulated by 0.26~1.91 (log2; FDR < 0.05), especially the highly expressed DEGs encoding y-type HMW-GSs, ω-gliadin and α-gliadin. These results were basically consistent with the result of RP-HPLC analysis.

**Table 5:** Log2 values of DEGs (N_0_ VS N_225_) encoding GSPs in developing grain.

ID	10 d	15 d	20 d	25 d	30 d	35 d	MEL	Description
Traes_1AS_AA6B839C5	−0.17	**1**.**10**^c^	**0**.**53**	**0**.**97**	**0**.**74**	**2**.**27**	622	12S seed storage globulin 1
Traes_1AS_E8827D028	−0.25	**1**.**32**	**0**.**60**	**0**.**88**	**0**.**58**	**2**.**35**	169	12S seed storage globulin 1
Traes_1DS_4426B6725	0.10	**0**.**85**	**0**.**79**	**1**.**21**	**1**.**13**	**2**.**20**	1696	Triticin
XLOC_005058	0.51	**0**.**48**	**0**.**51**	**0**.**80**	**0**.**82**	**1**.**11**	212	Globulin
Traes_4BS_4E68C8E47	1.88	**−0**.**63**	−0.27	0.21	−0.36	0.02	1109	Globulin 3
Traes_4BS_994BC287F	1.31	**−0**.**60**	−0.26	0.09	−0.16	−0.11	1847	Globulin 3
Traes_4DS_C2E603176	1.24	**−0**.**83**	−0.16	0.10	−0.28	−0.06	2356	Globulin 3
XLOC_109977	0.54	**−0**.**35**	−0.20	0.22	−0.30	−0.12	1483	Globulin 3
XLOC_115404	0.52	−0.09	−0.15	0.16	−0.25	**−0**.**31**	2162	Globulin-1 S allele
Traes_4AL_3D862C090	0.74	**−0**.**55**	−0.21	0.07	−0.23	−0.19	5148	Globulin-3A
XLOC_082451	0.14	−0.02	0.10	0.05	0.18	**0**.**29**	32540	LMW-GS
Traes_1DS_66B67E9B41	0.26	0.20	**0**.**39**	0.01	−0.03	**0**.**50**	5992	LMW-GS
Traes_1DL_D861501F5	0.23	**0**.**26**	0.12	**0**.**61**	0.22	**0**.**91**	3759	y-type HMW-GS
Traes_4AL_4FF5B8837	0.04	**0**.**77**	**0**.**34**	**0**.**45**	0.64	**0**.**78**	14861	α-gliadin
Traes_4AL_661613B77	−0.29	**0**.**50**	0.02	**0**.**47**	0.69	**0**.**96**	2489	α-gliadin
Traes_5BL_E68C461B3	0.54	**0**.**62**	**0**.**42**	**0**.**61**	**0**.**70**	**1**.**45**	6421	α-gliadin
XLOC_090782	**0**.**43**	**0**.**49**	**0**.**34**	**0**.**40**	**0**.**53**	**0**.**78**	11342	α-gliadin
XLOC_001662	−1.32	**−1**.**35**	−0.24	**2**.**68**	**−1**.**17**	**0**.**70**	93	γ-gliadin
Traes_1DS_67B7153A8	0.32	0.22	**0**.**41**	0.16	**0**.**44**	**0**.**65**	18454	Gliadin/avenin-like seed protein
Traes_4AL_FC0B3C3D31	0.04	**−0**.**55**	−0.22	**−0**.**50**	**−0**.**75**	0.14	8130	Gliadin/avenin-like seed protein
XLOC_003782	0.16	**0**.**80**	0.44	**1**.**24**	**0**.**93**	**1**.**91**	502	ω-gliadin
XLOC_003911	0.23	**1**.**03**	**0**.**81**	**1**.**34**	**0**.**66**	**1**.**44**	779	ω-gliadin

N_0_ VS N_225_, comparison of gene expression under N_225_ treatment to that under N_0_ treatment. MEL indicates the mean of expression level (FPKM value) during 10~35 DAA. The bold indicates FDR < 0.05.

### DEGs encoding transcription factor

Twenty six DEGs encoding transcription factor were found with at least one fold change (log2, FDR < 0.05), including 11 up-regulated and 15 down-regulated (Table 6). Four, two and two of up-regulated DEGs were responsible for encoding NAC, MYB, MADS-box transcription factors, respectively. The four DEGs encoding NAC appeared during 25~35 DAA. Three and four of down-regulated DEGs were in charge of encoding bHLH and WRKY transcription factors, respectively, and their expression were remarkably altered during 10~25 and 15~35 DAA, respectively.

**Table 6:** Log2 values of DEGs (N_0_ VS N_225_) encoding transcription factor during 10~35 DAA.

ID	10 d	15 d	20 d	25 d	30 d	35 d	Description
Traes_7BL_0459A249B	**1**.**25**	−0.39	−0.95	−0.82	−0.13	−0.09	Heat stress transcription factor A-3
XLOC_038196	**2**.**38**	−0.33	0.25	−0.47	0.45	−0.21	MADS-box transcription factor 29
Traes_1AS_106ED8AD4	−0.05	−0.06	0.17	0.06	0.37	**1**.**13**	MADS-box transcription factor 58
XLOC_069996	0.17	−0.08	−0.04	0.04	0.13	**1**.**01**	NAC transcription factor NAM
TRAES3BF020100130CFD_g	0.17	−0.12	−0.10	0.87	0.42	**1**.**08**	NAC transcription factor NAM
Traes_7BS_CF3224FD0	0.48	−0.02	−0.16	0.16	−0.06	**1**.**24**	NAC transcription factor NAM
XLOC_077043	0.71	0.79	−0.06	**1**.**71**	0.99	**2**.**35**	NAC transcription factor NAM
TRAES3BF171600010CFD_g	1.39	**1**.**40**	−0.12	0.20	−0.46	0.09	Ethylene-responsive transcription factor ERF071-like
Traes_4BL_545A5716E	−0.22	**0**.**57**	0.12	0.51	0.10	**1**.**11**	Transcription factor MYB44-like
Traes_3AL_E3BF20F0D	−0.09	**1**.**60**	1.22	0.00	0.00	0.00	Transcription factor MYB86
Traes_2BL_7CEC6A8D7	−0.90	0.52	−0.08	0.27	−0.18	**1**.**26**	Transcription factor LAF1
Traes_4BL_5D316DD23	0.78	1.14	0.08	0.48	−0.42	**−1**.**27**	AP2-like ethylene-responsive transcription factor AIL1
Traes_5BL_2D50EC294	**0**.**29**	−0.28	0.06	−0.14	−0.04	**−1**.**10**	bzip-related transcription factor -like
Traes_5BS_49C7C1976	0.25	−0.44	−0.25	−0.17	0.10	**−1**.**00**	CCAAT-binding transcription factor B
Traes_6AS_4B9F0CBBF	−0.17	0.18	−0.06	0.85	−0.33	**−1**.**80**	Transcription factor KAN2]
Traes_2BS_CD0E277C1	−0.32	**−1**.**58**	0.25	−0.30	−0.20	−0.56	Transcription factor bHLH148
Traes_5DL_5DC0E1715	−0.16	−0.27	−0.26	**−1**.**00**	−0.22	−0.34	Transcription factor bHLH150-like
Traes_1BS_FF6ECA0DB	**−2**.**53**	1.11	−0.58	0.88	−0.90	−0.06	Transcription factor bHLH68
Traes_7BS_F67ED5C9E	−0.67	−0.92	−0.79	−0.48	0.09	**−1**.**93**	Transcription factor SPATULA-like
Traes_6DL_DBD2A14D3	0.08	−0.28	0.25	**−1**.**10**	−0.20	−0.11	Transcription factor GTE9
Traes_4DS_9C2FD9F381	**−1**.**09**	0.11	0.07	0.75	−0.68	0.93	Transcription factor PCF3, partial
Traes_2AS_3B7BE6FDC	−0.21	0.05	0.62	0.27	0.73	**−2**.**64**	Transcription factor RF2b
Traes_2BS_B65714572	0.73	**−1**.**26**	−0.99	−0.55	0.66	−1.10	WRKY transcription factor WRKY1A
Traes_1BS_EF67E5A24	0.17	**−1**.**76**	1.98	−0.47	−0.49	0.22	WRKY14 transcription factor
Traes_3AL_0C7CB044E	1.20	**−2**.**17**	−0.75	−1.58	−0.31	0.41	WRKY27 transcription factor
Traes_3B_990298FF5	0.24	**−1**.**04**	0.30	−0.78	0.07	**−0**.**97**	WRKY27 transcription factor

The bold indicates FDR < 0.05.

## Discussion

**Figure 6:**
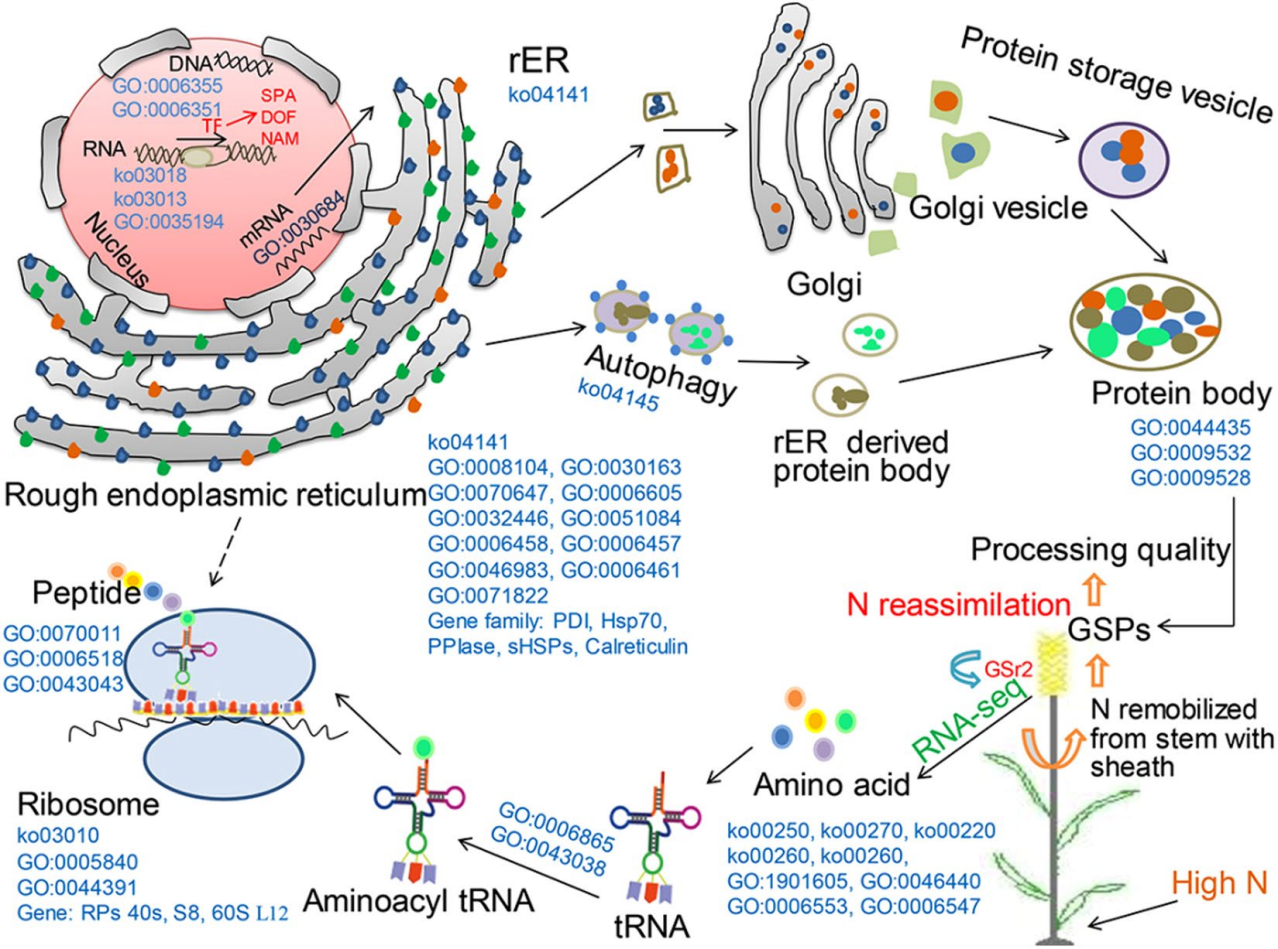
A putative mechanism of storage protein deposition in grains affected by N application. GSPs, grain storage proteins; rER, rough endoplasmic reticulum; RPs, ribosome proteins.

N application is considered as the most important agronomic input due to its contribution to processing quality of wheat, whereas the mechanisms on the contribution of N remain unclear. In the current study, a combination of physiological and transcriptomic analysis was used to identify the basis to partition N into GSPs in response to increased N inputs during grain development, and the putative mechanism based on our research data was summarized in Fig. 6.

N application has a beneficial effect on GSPs and its fraction in flour^15,16^. Similar result was observed in the present study. As for GSPs fraction, HMW-GSs and ω-gliadin, especially ω-gliadin, greatly responded to increased N by increasing their accumulation in grain^19,20^. In our experimental conditions, the elevated percentage was the lowest for ω-gliadin, and that was the highest for HMW-GSs at both sites. Meanwhile, principal components analysis indicated that the contribution of HMW-GSs was the highest among GSPs fractions on PC1 axis, consistent with previous result^17^. Taken together, HMW-GSs accounted for the largest effect of N application on GSPs factions, which was possibly responsible for the dominant increase in the ratio of HMW-GSs to LMW-GSs, thereby resulting in pronounced variation in wheat processing quality.

It is well accepted that a large number of grain N is derived from the remobilization of pre-anthesis absorbed N in vegetative organ. Although approximately half of the mobilized N originated from leaf blades, comparable difference happens in elsewhere, in stem or sheath^8,13^. In our experimental conditions, GSPs fractions and processing quality showed strong correlations with the mobilized N from stem with sheath, rather leaf blades or spike, suggesting that the altered GSPs accumulation and processing quality after N application were largely explained by the mobilized N from stem and sheath. Therefore, it would be valuable to pay more attention to the mobilized N from stem and sheath in future programs for wheat breeding. Only GI among these processing quality parameters exhibited clear relationships with the mobilized N from all vegetative organs (Table 4). GI was positively associated with the mobilized N from spike, while negatively associated with that from leaves, stem and sheath. Hence, it was reasonable to speculate that GI was a combined result of the mobilized N from all vegetative organs. The capacity of N remobilization can be inhibited by excess N^21^. Here, only N remobilization from spike was inhibited by increased N application to some extent (Fig. 2), thereby breaking the homeostasis of composition in GSPs fractions, and eventually leading to the decrease of GI. This observation indicates that the capacity of N remobilization in spike prior to other vegetative organs is repressed by N.

GS, consisting of the cytosolic (*GS1*) and chloroplastic (*GS2*), plays an essential role in plant nitrogen metabolism for its responsibility to assimilate ammonium and transform it into glutamine at the first step in GS and GOGAT cycle^22,23^. *GS1*, including *GS1* (*a*, *b* and *c*), *GSe* (1 and 2), and *GSr* (*1* and *2*), contributed more than *GS2* to the reassimilation of N^10^. Numerous studies focused on the role of GS enzyme in leaf, whereas its role in grain remained unclear. QTL analysis indicated that *GS1* was associated with grain size^24^, and *GS2* was co-localized with the locus for GPC^22^. Furthermore, GPC was positively relevant to the activity of GS in leaf^10^, and GS activity in leaf was significantly improved by N application^13,14^. GS activity in developing grain and GPC were positively correlated with N level as well in this research, which was supported by RNA-Seq and qRT-PCR data (Table S5 and Fig. 4). We demonstrated that ammonium reassimilation remained active in grain, thereby providing additional supply of amino acids after N application.

GSPs synthesis needs abundant supply of amino acids, which are principally transported from vegetable organ via phloem and tightly associated with signal transduction between nutrient availability and GSPs accumulation^25^. Increasing N level induced coordinated up-regulation of genes involved in the metabolism of alanine (Ala), glutamate (Glu), phenylalanine (Phe), tyrosine (Tyr) and tryptophan (Trp), but it down-regulated some genes responsible for aspartate metabolism in grain during filling stage. Importantly, DEGs encoding Tyrosyl-tRNA synthetase, Alanyl-tRNA synthetase, Valyl-tRNA synthetase, Glutaminyl-tRNA synthetase exhibited up-regulation during early and late filling stage (Supplementary Table S6). These consistent evidences manifested that the transport and synthesis of Glu, Val, Ala and Tyr responded to N application, resulting in increased accumulation of GSPs. Meanwhile, it was worthy to note that the diminished DEGs encoding asparaginase at high N level might lead to the decrease of asparagine that generate the carcinogen acrylamide during the baking process in mature grain^26^. It suggested that nitrogen application was beneficial to produce more healthy wheat flour, and the adjustment in amino acid metabolism was necessary to adapt GSPs accumulation in response to N application.

GSPs are synthesized on the rough endoplasmic reticulum (rER) (Fig. 6). Firstly, mRNA specially binds to ribosomes on the rERs, thereby forming polypeptide chains, which are the precursors of GSPs. Subsequently, these precursors of GSPs are transferred into the lumen of the rERs where lots of post-translational processes happened, such as cleavage of signal peptide, formation of intra- and inter-chain disulphide bonds, folding, assembly and aggregation of polypeptides. Finally, gliadins and glutenins from rERs are deposited into protein bodies (PBs) in the vacuole of endosperm cell via the Golgi apparatus and autophagy, respectively^27^. These processes are associated with rER-localised chaperones and foldases, which are divided into three groups, protein disulphide isomerases (PDI), peptidyl-prolyl cis-trans isomerases (PPIase), and the binding protein (BiP)/heat shock protein (Hsp70) chaperone^27–29^. According to KO enrichment analysis, we found that protein processing at rER and ribosomes were very active. *PDI* and *HSP70* genes were down-regulated by high N supply during mid-filling stage (Supplementary Table S6). Meanwhile, the genes coding for the 15~30 kDa small heat shock proteins (sHSPs) and the molecular chaperones that block the aggregation of unfolded proteins^30^, expressed as *PDI* and *HSP70*. Therefore, it was conceivable that rER responded to N starvation by up-regulation of *PDI*, *BiP* and *sHSPs* family during mid-filling stage, thereby enhancing the processing and accumulation of GSPs, and that might lead to high N use efficiency. On the other hand, PPIase gene family including *PPIase Pin1* and *FK506-binding protein 2* (FKBP)^31^, showed up-regulation during early and late filling stage. Moreover, *BAG2*, and *DnaJ8*, encoding co-chaperone that assist HSP70 to carry out chaperone activity^32,33^, as well as *calreticulin* that possesses molecular chaperone activity in protein folding^34^, were up-regulated during late filling stage. Although PPIases and co-chaperone of HSP70 were often ignored during protein folding in previous studies, they together with calreticulin were crucial for facilitating GSPs synthesis to cope with excess amino acids at rER under high N condition, particularly during early and late filling stage. Ribosomal proteins (RPs) are the major component for ribosome and play crucial roles in protein synthesis. Our study showed that most of RPs genes were repressed at high or low N level in certain period, especially *RPs 40s S8* and *60S L12*, which exclusively expressed at low N level during 10~20 DAA and at high N level during 25~35 DAA. Consequently, we speculated that ribosomal structure located at rER might change with N supply in grains, and *RPs 40s* and *60S* were good candidate genes for respecting N status.

In this study, the expression level of DEGs encoding GSPs was basically consistent with its accumulation except γ-gliadin, which has minor effect on properties of wheat flour^35^. The expression of GSPs genes are modulated by specific transcription factors (TFs) in response to N supply. SPA (Storage protein activator) and DOF (DNA binding with one finger) was a generally accepted TF for GSPs^27^, and they was up-regulated under sufficient N condition during early and late filling stage. In addition, several remarkably upregulated TFs (*NAM*, *MADS-box 29* and *58*, *MYB 44* and *86*) during mid and late filling stage, were also possibly involved in the transcriptional regulation of GSPs genes subjected to high N treatment. NAC, one of super TF family in plant, was highlighted for direct relation with leaf senescence and N allocation to grain during grain filling^36,37^, due to consisting of A to E domains for DNA-binding and protein-protein interactions^38^. However, little is known about its role in developing grain. MADS-box genes, with a highly conserved 180-bp-long motif, were emphasized curial roles in flower development and organ differentiation^39^. A MADS-box gene *SlFYFL* was proved to delay senescence, fruit ripening and abscission in tomato^40^. MYB TFs, with the presence of 1~4 or more imperfect MYB repeats domain located near the N-terminus, were vital regulators and modulate diverse biological processes, including plant response and tolerance to the nutrient deficiency^41^. Collectively, these TFs could serve as promising candidate genes for wheat quality breeding and deserve further research.

## Conclusions

The establishment of wheat processing quality was significantly affected by N fertilizer. Sufficient N supply promoted the remobilization of pre-anthesis absorbed N in vegetative organ and even N reassimilation governed by GS enzyme in grains, resulting in increased accumulation of GSPs. Meanwhile, the high-throughput RNA-seq analysis allowed us to investigate an overall survey of genes and processes potentially related to the response of GSPs to stimuli induced by high N level, which were mainly involved in adaptation in amino acid metabolism, protein processing at ER, transcriptional regulation for GSPs genes. Therefore, these comprehensive observations deepen our understanding in the mechanisms of GSPs in response to N nutrient and optimization of N strategy for wheat cultivar with superior quality.

## Materials and Methods

### Wheat cultivars and field experiments

Four locally adapted common wheat (*Triticum aestivum* L.) cultivars, including medium-gluten wheat cultivars, Shumai 969 (SM969) and Shumai 482 (SM482), and weak-gluten cultivars, Chuannong 16 (CN16) and Mianmai 51 (MM51), were grown at Chongzhou (30.5°N 103.7°E, sub-humid region) and Renshou (30°N 104.2°E, semi-arid region) experimental stations of Sichuan Agricultural University during two wheat growing seasons (2014.10–2015.5 and 2015.10–2016.5). Environmental condition was detailed in Supplementary Table S7. The soils at Chongzhou and Renshou experimental site were dark brown clay and purple sandy loam, respectively. The basic soil properties in 0~20 cm layer were given in Supplementary Table S8.

The four cultivars were evaluated at four levels of N supplies (urea), i.e. 0, 75, 150 and 225 kg ha^−1^ (referred to as N_0_, N_75_, N_150_ and N_225_, respectively). The field experiment was laid out as a split-block design with three replicates. Each individual plot size was 2 × 5 m at Chongzhou and 1.8 × 4.5 m at Renshou. Seeds were sown at 250 seeds m^−2^ with 20 cm row spaces in both field spots. The application of N as urea was spilt into 60% before sowing and 40% at stem elongation with immediate irrigation. A prophylactic programme for disease, weed, and pest management was used to maintain undisturbed healthy crop growth. Grains were harvested during mid-May at Chongzhou and early May at Renshou.

The flowering spikes, blooming on the same day, were marked with colored line, and the grains from middle ear at 10, 15, 20, 25, 30 and 35 days after anthesis (DAA) were collected at 2:00–3:00 pm. The collected samples were immediately frozen in liquid N_2_ and then stored at −80 °C.

### Evaluation of processing quality

Total plot was harvested and threshed using a mini-Vogel machine for recording yield. Two kilos of seeds were randomly picked from each grain sample to evaluate grain processing quality. Grain samples were stored for three months before milling. Milling was done on a Brabender Quadromat Juniors® (Brabender GmbH & Co. KG, Germany). Samples were conditioned to 14.0% moisture before milling. The grain protein content (GPC), zeleny sedimentation value (SV), wet gluten content (GC) and gluten index (GI) were determined following GB/T 17320–2013, by an automatic azotometer (Kjelec 8400; FOSS, Denmark), a zeleny analysis system (CAU-B, China) and a glutomatic 2200 system (Perten, Sweden), respectively. Dough rheological properties were tested by Farinograph®-AT (Brabender GmbH & Co. KG, Germany) with Mixer*50.

### Determination of the contents of gliadin and glutenin fractions

For each sample, 45 mg flour was used to extract gliadins and glutenins. The gliadin and glutenin fractions were analysed according to previous method^42,43^ with minor modifications, respectively. The gliadin and glutenin fractions were both filtered through 0.45 µm nylon filter (Teknokroma, Barcelona, Spain) before performing Reverse-phase high-performance liquid chromatography (RP-HPLC) analysis. Gliadin and glutenin extracts were applied to a ZORBAX 300SB-C18 reverse phase analytical column (4.6 × 250 mm, 5-Micorn; Agilent Technologies, Santa Clara, CA) using a 1100 Series Quaternary LC System liquid chromatograph (Agilent Technologies, Santa Clara, CA) with a DAD UV-V detector. Absorbance was monitored with the DAD UV-V module at 210 nm. The major analytical parameters were set as 60 °C for column temperature, 1.00 ml/min for flow rate, 20 µl for sample volume, eluting gradient and the variable concentrations of acetonitrile with 0.06% trifluoroacetic acid for gliadins and glutenins gradually growing from 21% to 48% (v/v) in 55 min and from 25% to 48% (v/v) in 50 min, respectively. Column washing time for gliadins and glutenins between two adjacent samples were 15 and 10 min, respectively. The integration procedure was handled automatically by the Agilent Technologies Chemistry Station software with minor manual adjustment. The amounts of HMW-GSs, LMW-GSs and gliadins were estimated by integrating the relevant peak areas in the chromatograms.

### Measurement of the ratio of glutenins to gliadins

Albumins, globulins, gliadins and glutenins were sequentially extracted using distilled water, 10% NaCl, 70% ethyl alcohol and 0.2% NaOH, respectively. After adding extracting solution, the mixture was shaken for 30 min at 220 rpm, and was then centrifuged for 15 min at 4000 rpm. The supernatant was extracts, and the precipitate was resuspended with next extracting solution. Every extraction step was repeated for three times. Protein content in the supernatant was tested with an automatic azotometer (Kjelec 8400; FOSS, Denmark). The ratio of glutenins to gliadins (Glu/Gli) was calculated by dividing their contents.

### Measurement of glutamine synthase (GS) activity

Fresh grains at 10, 15, 20, 25, 30 and 35 DAA were used to analyze changes in GS activity. All operations were performed at 4 °C. The activity was assayed following a reported method^44^ with minor modification.

Frozen grain powder (1 g) was suspended in 50 mM Tris-HCl buffer (pH 8.0, 4 ml) with 2 mM MgSO_4_.7H_2_O, 2 mM DTT and 400 mM sucrose. The extract was centrifuged for 20 min at 15,000 g at 4 °C. The supernatant was used as enzyme extract. The reaction medium (1.6 ml) contained 100 mM Tris-HCl (pH 7.4), 80 mM MgSO_4_.7H_2_O, 20 mM L-glutamate-Na, 20 mM cysteine, 2 mM EDTA, 80 mM hydroxylamine. The reaction was started by the addition of 40 mM ATP-Na_2_ (0.7 ml) and enzyme extract (0.7 ml), and it was incubated for 40 min at 37 °C. The enzyme reaction was terminated by adding 1 ml of 370 mM FeCl_3_ and 200 mM trichloroacetic acid in 600 mM HCl. The reaction mixture was centrifuged for 10 min at 12,000 g at 25 °C. GS activity was measured by spectrophotometer at 540 nm and expressed as A540 g^−1^ FW h^−1^.

### Measurement of N content in vegetative organs

Twenty plants of each plot were collected at anthesis and maturity. Plants were divided into flag leaf blade, lower leaf blades, stem with sheath and spike. Fresh samples were immediately treated in an oven at 105 °C for 45 min and then dried at 75 °C until they reached a constant weight. Dry samples were milled into flour for automatic azotometer analyses as above (Kjelec 8400; FOSS, Denmark).

### RNA-seq analysis

Transcriptional analysis was only carried out in whole grain samples of SM482 under N_0_ and N_225_ treatments, collected at 10, 15, 20, 25, 30 and 35 DAA at Chongzhou site during 2014–2015 growing season. There were two biological replicates for grain sample at each stage. The midsection of ear with grain was freeze-dried in liquid N_2_, and grains were stored at −80 °C for RNA extraction. Grains were ground into find powder in liquid N_2_ and total RNA was extracted as described by Plant RNA extraction kit V1.5 (Biofit, China, http://www.biofit.com). Three µg RNA per sample was used as input material for the RNA sample preparation. The mRNA samples were enriched by using oligo (dT)-magnetic beads and then cut into fragments with fragmentation buffer at 80 °C. First-strand cDNA was synthesized with random hexamers using the fragments as templates. Second-strand cDNA was subsequently performed using RNase H, DNA polymerase I, and dNTPs. The cDNA was purified by QiaQuick PCR kit and eluted with EB buffer. After terminal repair, poly A and adaptor sequences were connected to the cDNA end. cDNA libraries were established by PCR amplification after screening fragment size and assessing quality on Agilent 2100 Bioanalyzer system. The qualified cDNA libraries were sequenced on Illumina HiSeq2500^TM^ system at Gene Denovo Co., Ltd. (Guangzhou, China).

Clean data (clean reads) were obtained by removing reads containing adapter, reads containing ploy-N and low quality reads from raw data. The clean reads were matched to the *T*. *aestivum* cDNA database from ensemblgenomes (ftp://ftp.ensemblgenomes.org/pub/release-28/plants/fasta/triticum_aestivum/cdna/Triticum_aestivum.IWGSC1.0+popseq. 28.cdna.all.fa.gz, released on 30 July 2015) using TopHat v2.0.12/Bowtie v2.2.3 software. DEGs were screened using a log2-fold change at *P* < 0.05 and FDR (padjust) <0.05 level. Gene ontology (GO) and Kyoto Encyclopedia of Genes and Genomes (KEGG) pathway analysis were performed to investigate DEG functions. The fragment per kb per million reads (FPKM) value was utilized to calculate gene expression level.

### Quantitative real-time PCR

To validate the reliability of expression profiles in RNA-seq data (N_0_ VS N_225_), 10 genes were selected for quantitative real-time PCR (qRT-PCR) analyses, using a SYBR premix Ex Taq^tm^ RT-PCR kit (Takara, Dalian, China). Primers were designed by Primer Premier 5.0 (Premier Biosoft, Palo Alto, Canada) and listed in Supplementary Table S9. *GAPDH* (Genbank accession number KU246046.1), *TUBA*-2A (DQ435659.1), and *TEF1* (M90077.1) were used as reference. The relative expression value was calculated by the ∆∆Ct method.

### Statistical analysis

All the data were calculated by using Excel 2010 (Microsoft, USA). Analysis of variance (ANOVA) was performed using the DPS software version 12.01^45^. Least significant difference (LSD) was used to compare difference among experimental mean values (*P* < 0.05). A bivariate correlation procedure was performed to analyze the relationships between these traits.

## Electronic supplementary material

Supplementary Table S1, S5-S9 and Figure S1

Supplementary Table S2

Supplementary Table S3

Supplementary Table S4
