# Supplementary material for: Mechanisms of wheat (Triticum aestivum) grain storage proteins in response to nitrogen application and its impacts on processing quality
Source: Sci Rep. 2018 Aug 9;8:11928. doi: 10.1038/s41598-018-30451-4 (PMC6085318; doi:10.1038/s41598-018-30451-4)
Supplement: Supplementary file 1 — Supplementary Table S1, S5-S9 and Figure S1 [file 41598_2018_30451_MOESM1_ESM.pdf]

Mechanisms of wheat (*Triticum aestivum*) grain storage proteins in response to nitrogen application and its impacts on processing quality

Ting Zheng[1,+] Peng-Fei Qi[1,+,*], Yong-Li Cao[1], Ya-Nan Han[1], Hong-Liang Ma[2], Zhen-Ru Guo[1], Yan Wang[1], Yuan-Yuan Qiao[1], Shi-Yu Hua[2], Hai-Yue Yu[2], Jiang-Ping Wang[1], Jing Zhu[1], Cai-Yi Zhou[1], Ya-Zhou Zhang[1], Qing Chen[1], Li Kong[1], Ji-Rui Wang[1], Qian-Tao Jiang[1], Ze-Hong Yan[1], Xiu-Jin Lan[1], Gao-Qiong Fan[2], Yu-Ming Wei[1], and You-Liang Zheng[1,*]

[1] Triticeae Research Institute, Sichuan Agricultural University, Chengdu, Sichuan 611130, China

[2] Agronomy College, Sichuan Agricultural University, Chengdu, Sichuan 611130, China

[+] Contributed equally to this paper

[*] Corresponding authors. P.-F. Qi, E-mail: pengfeiqi@hotmail.com; Y.-L. Zheng, E-mail: ylzheng@sicau.edu.cn; Phone: +86-28-82650337; Fax +86-28-82650350

Traits	PC1	PC2	PC3	PC4
HMW mAU	0.372	0.114	-0.233	0.332
LMW mAU	0.051	0.457	-0.204	0.323
H/L	0.286	-0.341	0.080	-0.023
Glutenins mAU	0.190	0.390	-0.248	0.378
ω- mAU	0.349	-0.286	-0.085	0.103
α/β- mAU	0.345	0.200	0.012	-0.433
γ- mAU	0.372	0.183	0.284	-0.211
Gliadins mAU	0.409	0.084	0.082	-0.265
ω- %	0.181	-0.431	-0.142	0.239
α/β- %	-0.285	0.275	-0.278	-0.382
γ- %	0.126	0.292	0.633	0.174
Glu/Gli	-0.255	-0.022	0.492	0.310
Eigenvalues	5.25	3.92	1.15	0.96
% Variance	43.71	32.68	9.62	8.04
Sum % variance	43.71	76.40	86.02	94.06

Table S1. The first four principal components for RP-HPLC Data, eigenvalues and % of variance explained by PCs. LMW, low-molecular-weight glutenin subunits; HMW, high-molecular-weight glutenin subunits; H/L, ratio of HMW to LMW; Glu/Gli, ratio of glutenins to gliadins; ω-%, α/β- % and γ- % indicate the percentages of ω-, α/β- and γ-gliadins of total gliadins, respectively.

ID	K_ID	Annotation	Pathway	S&FC
XLOC_085270	K00814	Alanine aminotransferase 2	Alanine, aspartate and glutamate metabolism; Arginine biosynthesis	35 d, 0.72
XLOC_006117	K00814	Alanine aminotransferase 2	Alanine, aspartate and glutamate metabolism; Arginine biosynthesis	35 d, 0.71
Traes_4DL_CB8E1061C	K01915	Glutamine synthetase isoform GSr2	Alanine, aspartate and glutamate metabolism; Arginine biosynthesis	35 d, 0.97
Traes_4AS_1CDA71553	K01915	Glutamine synthetase isoform GSr3	Alanine, aspartate and glutamate metabolism; Arginine biosynthesis	30 d, 0.98
Traes_4BL_52F044A40	K01915	Glutamine synthetase isoform GSr1	Alanine, aspartate and glutamate metabolism; Arginine biosynthesis	30 d, 0.72
Traes_2BL_0D44F4C96	K01953	Asparaginase	Alanine, aspartate and glutamate metabolism	25 d, -1.02
Traes_2BL_AE75D6172	K01953	Asparaginase	Alanine, aspartate and glutamate metabolism	15 d, -1.52
Traes_2BL_BDC074418	K01953	Asparaginase	Alanine, aspartate and glutamate metabolism	25 d, -1.06
Traes_4DL_06C6DB821	K01953	Asparagine synthetase	Alanine, aspartate and glutamate metabolism	15 d, 1.20
Traes_6DL_6DAB8F1D9	K00130	Betaine-aldehyde dehydrogenase	Glycine, serine and threonine metabolism	35 d, 1.00
Traes_2DL_1FF716E43	K00276	Primary amine oxidase	Glycine, serine and threonine metabolism	35 d, -1.09
Traes_2AL_CD28AB70E	K00277	Primary amine oxidase	Glycine, serine and threonine metabolism	35 d, -0.77
Traes_5DS_9C45AD986	K00547	Homocysteine S-methyltransferase 3	Cysteine and methionine metabolism	35 d, 1.30
Traes_5AS_9DEE71A2C	K00547	Homocysteine S-methyltransferase 3	Cysteine and methionine metabolism	35 d, 0.72
Traes_2AL_BE2D14989	K01251	Adenosylhomocysteinase	Cysteine and methionine metabolism	35 d, -0.90
XLOC_000237	K01919	Gamma-glutamylcysteine synthetase	Cysteine and methionine metabolism	15 d, -1.06
Traes_2DL_5247AB0F3	K00140	Methylmalonate-semialdehyde dehydrogenase [acylating], mitochondrial	Valine, leucine and isoleucine degradation; beta-Alanine metabolism	35 d, 0.77
Traes_2DS_E6C77EB38	K00249	Acyl-CoA dehydrogenase family member 10	Valine, leucine and isoleucine degradation; beta-Alanine metabolism	15 d,
Traes_2DL_071CC70F4	K10536	Agmatine deiminase	Arginine and proline metabolism	35 d, -0.79
Traes_1AL_014F29DA6	K12657	Delta 1-pyrroline-5-carboxylate synthetase	Arginine and proline metabolism	15 d, 1.01
Traes_4AL_2DC24CE38	K00001	Alcohol dehydrogenase ADH2A	Tyrosine metabolism	15 d, -1.24
Traes_6AL_67ED9DB4D	K13064	Phenylalanine ammonia-lyase 4	Phenylalanine metabolism	35 d, -0.96
Traes_2DS_E231054BE	K01626	Phospho-2-dehydro-3-deoxyheptonate aldolase 2, chloroplastic	Phenylalanine, tyrosine and tryptophan biosynthesis	35 d, 0.88
Traes_2AS_BEC9B768B	K01627	Phospho-2-dehydro-3-deoxyheptonate aldolase 3, chloroplastic	Phenylalanine, tyrosine and tryptophan biosynthesis	35 d, 0.80
Traes_2BS_B6E90913E	K01628	Phospho-2-dehydro-3-deoxyheptonate aldolase 4, chloroplastic	Phenylalanine, tyrosine and tryptophan biosynthesis	35 d, 0.80

Table S5. DEGs (N_0 VS N_{225}) involved in amino acid metabolism according to KEGG pathway database. DEGs were screened by FPKM>10, log2>0.7 and FDR<0.05. N_0 VS N_{225}, N_{225} compared to N_0. S&FC, expression stage (days post anthesis) and fold change (log2). K_ID, KEGG pathway ID.

ID	Annotation	S&FC
XLOC_054847	Alanyl-tRNA synthetase, cytoplasmic	10 d, 1.52
Traes_7DL_83B926810	Glutaminyl-tRNA synthetase	10 d, 1.37
XLOC_028369	Threonyl-tRNA synthetase, mitochondrial	10 d, 1.11
XLOC_056116	Tyrosyl-tRNA synthetase	10 d, 1.39
XLOC_073134	Tyrosyl-tRNA synthetase	10 d, 1.49
Traes_2DL_2FC93A5E5	Valyl-tRNA synthetase	15 d, 1.75
XLOC_052499	Peptidyl-prolyl cis-trans isomerase Pin1-like	10 d, 1.03
Traes_6AS_EC32B6D94	Protein disulfide isomerase-like 1-4	20 d, -10.46
Traes_3DS_E1BF0B967	16.9 kDa heat-shock protein	25 d, -1.17
Traes_1AL_D8322513E	16.9a kDa heat-shock protein	25 d, -1.09
Traes_4BL_D8322513E	16.9a kDa heat-shock protein	25 d, -1.09
Traes_3DS_BC0C261A51	16.9a kDa heat-shock protein	20 d, -1.42
Traes_3AS_396386369	17.0 kDa heat-shock protein	25 d, -1.02
Traes_3AS_552E74797	17.0 kDa heat-shock protein	25 d, -1.25
TRAES3BF066700080CFD_g	17.4 kDa heat-shock protein	25 d, -1.1
Traes_4BL_B02609A29	17.6 kDa class I heat shock protein	25 d, -1.1
Traes_2BL_33410A32A	Heat shock factor protein 4	25 d, -1.29
Traes_2BL_B2AE84667	Heat shock factor protein 4	25 d, -1.37
Traes_1BL_627D564A9	Heat shock protein	20 d, -1.66
Traes_3DS_909E445F1	Heat shock protein 16.9	20 d, -1.13
Traes_4BL_4D370C9FA	Heat shock protein 70	25 d, -1.45
Traes_4BL_4D370C9FA1	Heat shock protein 70	25 d, -1.45
Traes_5BL_9FDF53F78	HSP20 protein	20 d, -1.69
Traes_2BL_86887455D	FK506-binding protein 2-like	35 d, 1.14
Traes_2DL_447983A18	FK506-binding protein 2-like	35 d, 1.07
Traes_2DL_447983A18	FK506-binding protein 2-like	25 d, 1.19
XLOC_038403	Chaperone protein DnaJ	15 d, -2.39
XLOC_114051	Chaperone protein dnaJ 1, mitochondrial	10 d, -11.17
XLOC_058729	Chaperone protein dnaJ 10	10 d, 1.92
Traes_1AL_A77C27C53	Chaperone protein dnaJ 13	35 d, -4.05
Traes_4AL_A8C2B1294	Chaperone protein dnaJ 49	15 d, -11.74
Traes_7AL_E88CD714B	Chaperone protein dnaJ 8, chloroplastic	35 d, 1.51
Traes_7AL_E88CD714B	Chaperone protein dnaJ 8, chloroplastic	25 d, 2.43
Traes_7AS_28F6458D3	Iron-sulfur cluster co-chaperone protein HscB,	25 d, -2.30
TRAES3BF044000080CFD_g	BAG family molecular chaperone regulator 2-like	35 d, 2.37
TRAES3BF044000100CFD_g	BAG family molecular chaperone regulator 2-like	35 d, 1.57
XLOC_108420	BAG family molecular chaperone regulator 6 isoform X2	15 d, -1.15
Traes_5BL_01B2A4A96	Calreticulin	10 d, -1.50
Traes_5BL_DCBA0D416	Calreticulin	10 d, -2.28
Traes_5DL_7D83C2AB2	Calreticulin	35 d, 1.27

XLOC_000135	Calreticulin	10 d, 1.77
Traes_3AL_137E874E4	60S ribosomal protein L10a-1	15 d, 1.12
Traes_3AL_74778D9DF	60S ribosomal protein L10a-1	15 d, 1.13
XLOC_023242	60S ribosomal protein L12-1	35 d, -10.87
XLOC_059616	60S ribosomal protein L12	10 d, 11.90
XLOC_059707	60S ribosomal protein L12	20 d, 13.16
XLOC_059707	60S ribosomal protein L12	25 d, -13.10
XLOC_059707	60S ribosomal protein L12	30 d, -13.48
XLOC_059707	60S ribosomal protein L12	35 d, -14.83
XLOC_017739	60S ribosomal protein L28-1	10 d, 1.39
Traes_2BL_FFCF7A918	60S ribosomal protein L7-2	35 d, 1.25
Traes_2BL_FFCF7A918	60S ribosomal protein L7-2	20 d, 12.05
XLOC_010770	Ribosomal protein L16, partial (chloroplast)	35 d, -1.25
Traes_4DS_4C3C95D2E	Ribosomal protein L2 (chloroplast)	35 d, -12.14
Traes_5AL_4C3C95D2E	Ribosomal protein L2 (chloroplast)	35 d, -12.14
Traes_1DS_DABE8C075	Ribosomal protein L22 (chloroplast)	15 d, -11.93
Traes_6DS_DABE8C075	Ribosomal protein L22 (chloroplast)	15 d, -11.93
Traes_7DL_DABE8C075	Ribosomal protein L22 (chloroplast)	15 d, -11.93
Traes_3AL_1DF1A2927	Ribosomal protein L22 (chloroplast)	25 d, -12.15
Traes_6BL_30EFB4704	Ribosomal protein L17-like protein	20 d, -2.29
Traes_1BS_DD122AEA6	Ribosomal protein S18 (chloroplast)	15 d, -3.81
Traes_5DS_436DB5B2B	Ribosomal protein S18 (chloroplast)	10 d, -1.46
XLOC_012637	Ribosomal protein S20	15 d, 9.73
EPlTAEG00000010136	Ribosomal protein small subunit 3 (mitochondrion)	25 d, -1.16
Traes_6AL_F8B86A41E	Ribosomal protein S3 (chloroplast)	10 d, -12.21
Traes_7BL_41C34373C	Ribosomal protein S3 (chloroplast)	35 d, -3.78
Traes_4DL_6AE4D9044	Ribosomal protein S8 (chloroplast)	25 d, -12.28
XLOC_023152	40S ribosomal protein S27	10 d, 1.27
XLOC_063650	40S ribosomal protein S27	35 d, 1.05
XLOC_093297	40S ribosomal protein S8	20 d, 15.04
XLOC_093297	40S ribosomal protein S8	15 d, 13.15
XLOC_093297	40S ribosomal protein S8	30 d, -14.71
XLOC_093297	40S ribosomal protein S8	35 d, -14.32
Traes_4AL_BE1AD302B	30S ribosomal protein S5	35 d, -1.61
Traes_7DL_8D073D6DD	37S ribosomal protein MRP17, mitochondrial-like	35 d, -4.63

Table S6. DEGs (N_0 VS N_{225}) encoding amino acid transporter and DEGs involved in protein processing at Rough endoplasmic reticulum. N_0 VS N_{225} and S&FC are used as Table S5.

Month	Sunshine hours (h)		Average temperature (°C)		Highest temperature (°C)		Lowest temperature (°C)		Rainfall (mm)	
	Chongzhou	Renshou	Chongzhou	Renshou	Chongzhou	Renshou	Chongzhou	Renshou	Chongzhou	Renshou
October, 2014	53	71.9	18.1	19.5	22.1	23.0	15.5	17.1	75.5	87.3
November, 2014	32.6	40.7	12.1	13.5	15.6	16.4	9.8	11.8	18.6	19.4
December, 2014	69.5	60	6.2	8.4	11.1	11.7	2.7	5.9	5.7	8
January, 2015	25	21.1	7.1	9.0	10.9	11.7	4.3	6.9	3.9	8.9
February, 2015	59.2	64	8.8	11.1	14.0	14.9	5.1	8.3	2.7	9.4
March, 2015	71	106.3	13.3	16.0	18.7	20.7	9.6	12.6	9.4	11.9
April, 2015	130.5	158	17.7	20.5	24.0	25.9	13.2	16.1	90.3	66.1
May, 2015	207.2	212.3	22.0	23.8	28.2	29.4	17.4	19.7	86.7	65.6
October, 2015	67.7	71.3	18.4	19.9	23.2	24.0	15.3	17.3	25	26.8
November, 2015	27.8	31.4	13.8	15.4	17.0	18.0	11.5	13.7	14	20.6
December, 2015	46.2	28	7.2	9.5	11.3	12.2	4.4	7.2	13.2	12.4
January, 2016	53.4	47.9	5.6	7.2	9.9	10.6	2.6	4.6	13.6	16.4
February, 2016	109	109.3	7.7	10.3	14.3	15.5	3.1	6.4	35.4	42.1
March, 2016	64	63.6	12.6	14.9	18.4	19.8	8.0	12.1	34.6	40.7
April, 2016	84	75.1	17.5	19.3	23.1	24.1	13.5	15.6	66	65.2
May, 2016	108.5	125.4	20.5	22.6	26.1	28.0	16.5	18.3	94.7	93.4

Table S7. Meteorological conditions of the two experimental locations during 2014-2015 and 2015-2016 growing seasons.

Soil property parameter	Chongzhou	Renshou
Total N (g/kg)	1.95	1.23
Soil organic matter (g/kg)	49.61	32.41
Available N (mg/kg)	109	59
Olsen-P (mg/kg)	55.86	38.72
Exchangeable-K (mg/kg)	55.73	57.95
pH	7.34	7.59

Table S8. Basic soil properties in 0-20 cm soil layer before field trials at two experimental sites

Gene name	GenBank No.	Primers (5'-3')	
TUBA-2A	DQ435659.1	forward	TACCGTGGTGATGTTGTTC
		reverse	CTTGTGGTCAATGCGGGAG
GAPC1	KU246046.1	forward	AAGGCTGTTGGTAAGGTTC
		reverse	CCTGCTGTCACCAACAAAGT
TEF1	M90077.1	forward	AGGCTGACTGTGCTGTTC
		reverse	AGATGGGGACGAAGGGA
Wx-DI	AF163319.1	forward	GGACTACGAGGACAACCA
		reverse	GTAGATGCCATTGGACTG
BADH	AY050316.1	forward	TGCTTTGAGTTCTTCGC
		reverse	GGGGCTACCTTCCATACA
Sbe1D	AF286317.1	forward	ATAGTTTAGGGTTGCGTGTT
		reverse	ACAGGCGGCTATCCCAC
GLU4	JN128604.1	forward	AAGTATCAGCCTCACCAGA
		reverse	GGCAACCTGTTCCCGAC
NAM	HM027575.2	forward	AATGGGTAGTGCGGTGCTT
		reverse	CGTCCCAGTTAGAACATCCT
AGP-L	DQ839506.1	forward	CGACGACCCTGCTAAAT
		reverse	TCCAATGTCCTCCCAGTA
serpin-N3.2	JN903908.1	forward	GCGACGACTTCTACCTTCTTG
		reverse	TGGTTCGGCACTCAACTTTT
GSr2	AY491969.1	forward	GACATCAGGAGCAAAGCA
		reverse	ACGGGTCCTTGAAAATG
wSs2a-1	AJ269502.1	forward	TTGGCAGGGGAGAATGT
		reverse	CAGCCTTGTAGTATTTTCG
LOC109749392	XM_020308354.1	forward	TCCTCCTTCTCCAACACC
		reverse	AAGATACCAAAGGTGAAGG

Table S9. Primers used for qRT-PCR analysis

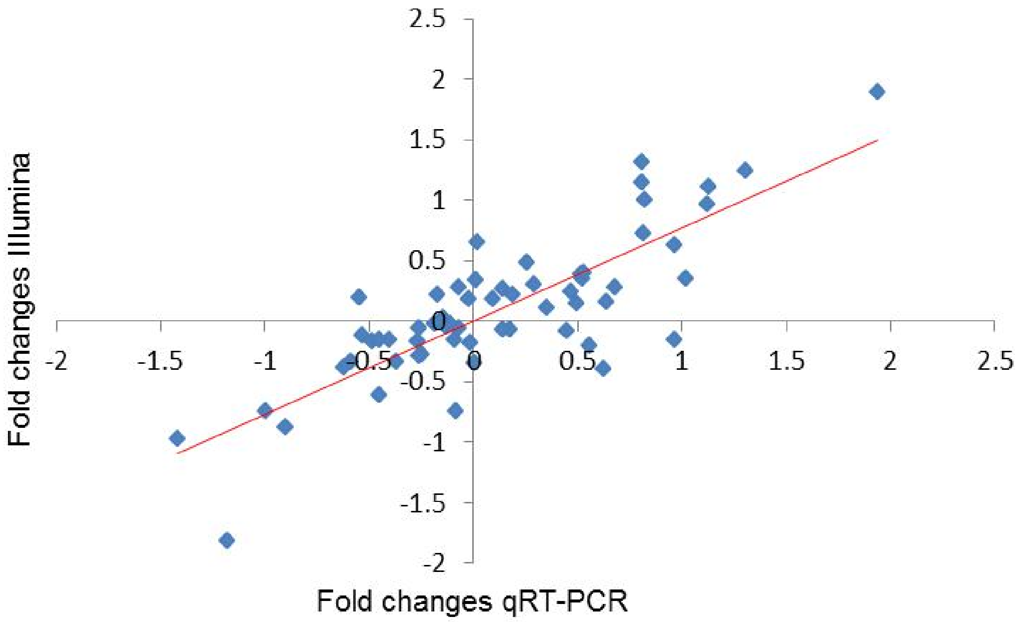

Figure S1. Correlation between the normalized RNA-seq results and qRT-PCR data.
